# Bilateral symmetrical stress fractures of femoral neck in HIV patient: case report and brief review of literature

**DOI:** 10.1259/bjrcr.20220003

**Published:** 2022-05-10

**Authors:** Ramakrishna Narra, Sravan Kumar Varra, Naganarasimha Raju Jukuri

**Affiliations:** 1 Department of Radio-diagnosis, Katuri Medical College, Guntur, India

## Abstract

Osseous changes in HIV positive patients receiving ART are three times greater than in normal individuals. These changes include osteopaenia/osteoporosis, stress fractures, fragility fractures and osteonecrosis. On an average, bone mineral density reduces by 2–6% in the first 2 years after commencement of antiretroviral therapy. High risk for stress fractures is observed in these patients. In present article, we report a case of bilateral femoral neck stress fractures in a known HIV patient.

## Clinical presentation

A 52-year-old gentleman presented to orthopaedic department with complaints of bilateral hip pain and limp for 1 month. He was diagnosed as HIV positive 12 years back and currently on HAART (Highly Active Anti-Retroviral Therapy) including dolutegravir, lamivudine and tenofovir disoproxil fumarate. The patient was on regular medication and follow-up. No history of any recent significant trauma to the hip was noted. He is a non-smoker and non-alcoholic and not a known case of diabetes mellitus. On clinical examination, unsteadiness of gait and tenderness in the bilateral hip region was noted. On routine blood examination blood urea level was 42.5 mg dl^−1^ (normal range 14–23 mg dl^−1^), and serum calcium level 11.8 mg dl^−1^ (normal range 8.6–10.3 mg dl^−1^) which were above the normal range. Rest of the blood examination and biochemical laboratory values were within normal limits.

### Investigations

**Figure 1.**
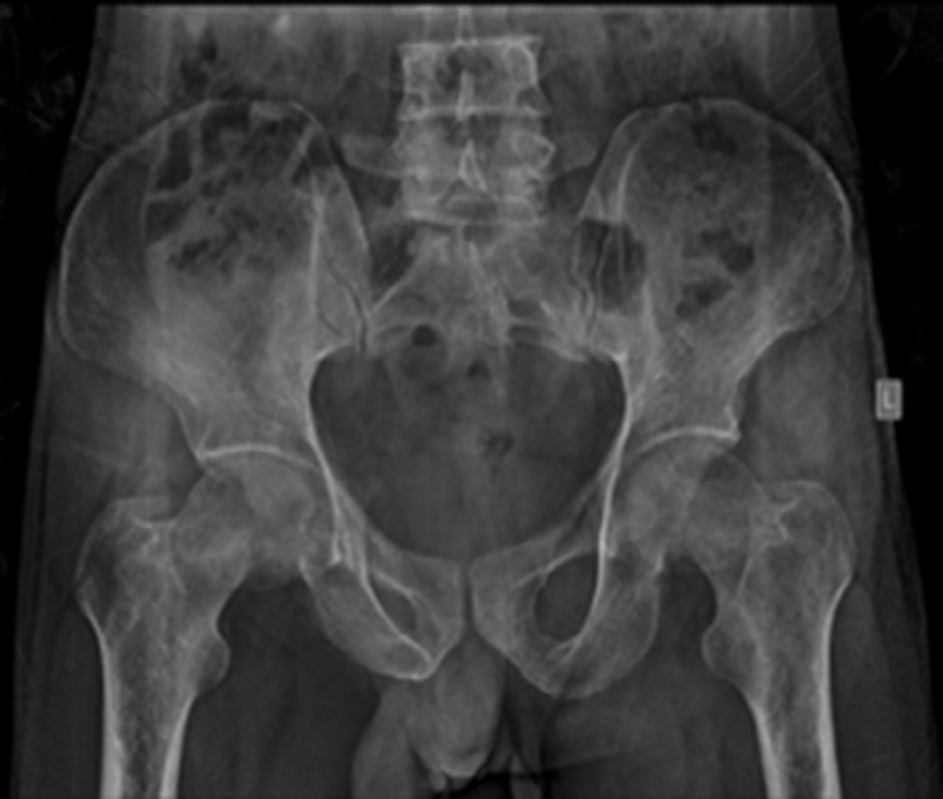
Anteroposterior radiograph of pelvis with bilateral hip demonstrating diffuse osteoporosis of the visualised pelvic bones and femur, with loss of continuity of the primary and secondary trabecula in the proximal femur bilaterally

Radiograph of the pelvis AP (Figure 1) performed demonstrated features of osteoporosis with partial loss of trabeculae in the proximal femur and visualised pelvic bones.

**Figure 2.**
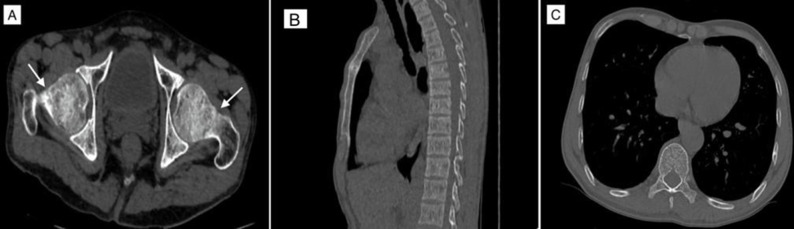
(A) CT scan of pelvis with bilateral hip demonstrating thin fracture line (arrows) in the bilateral femur neck with adjacent sclerosis suggestive of stress fractures. (B) Sagittal CT scan of dorsal vertebra demonstrating diffuse osteoporosis. (C) Axial CT scan at D7 vertebral level demonstrating osteoporosis as visualised by prominent thin trabecular pattern.

CT of pelvis and dorsolumbar spine (Figure 2) performed demonstrated linear thin fracture lines within the neck of both femurs (more prominent on the left side) and prominent vertical/primary trabeculae with thinned out horizontal/secondary trabeculae in vertebral bodies, loss of trabecular pattern, and thinning of the cortex suggestive of generalised osteoporosis with stress fractures in the neck of bilateral femur bones.

**Figure 3.**
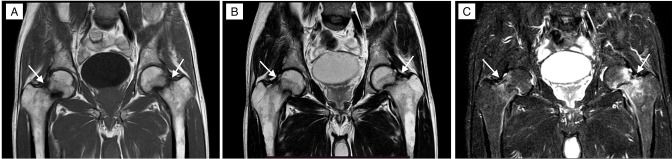
Coronal *T*_1_W (A), *T*_2_W (B), STIR (C) images demonstrating stress fractures in the bilateral femur neck as linear hypointense bands with surrounding oedema and sclerosis (Arrows). STIR, short-tau inversion recovery.

**Figure 4.**
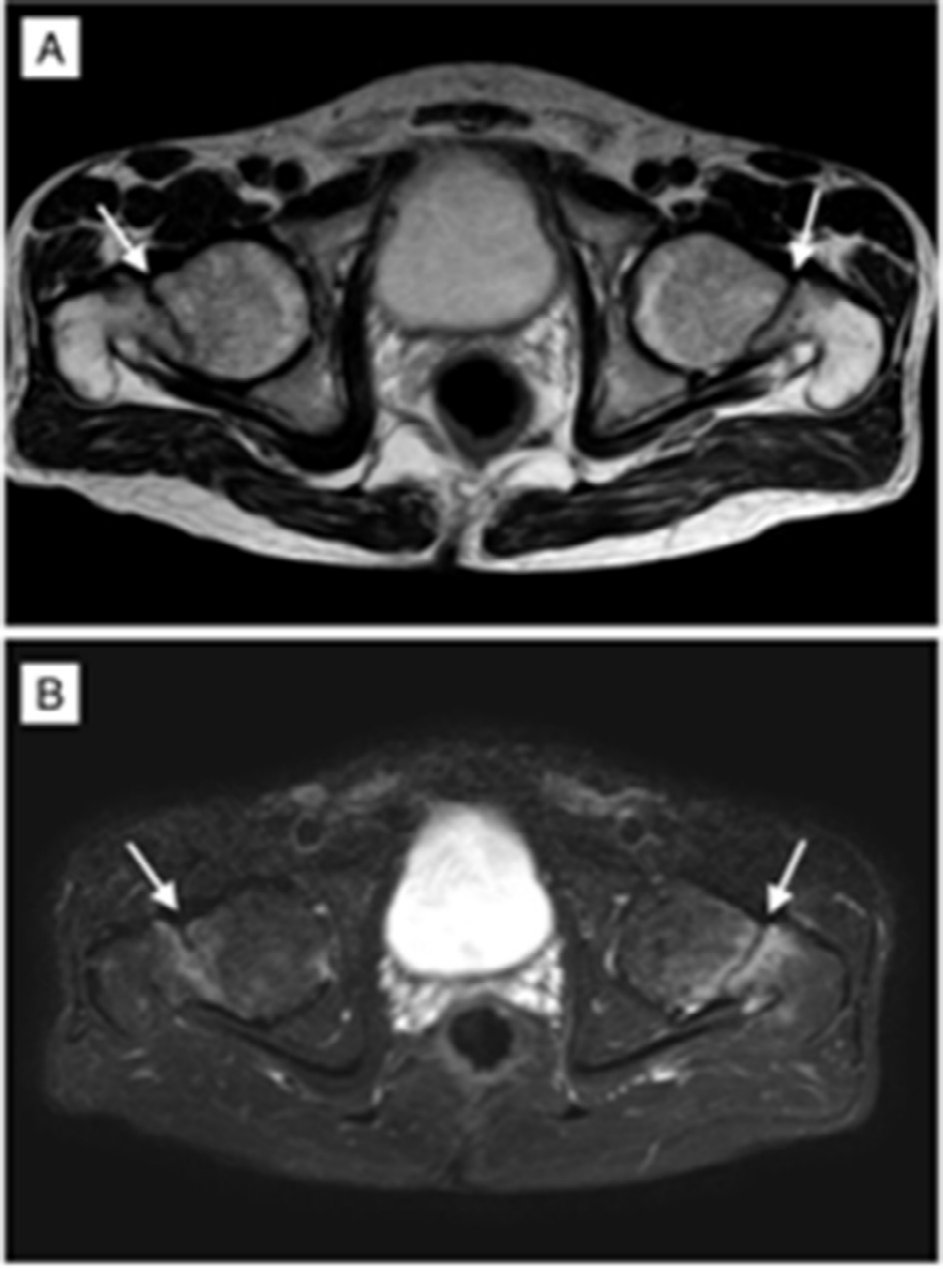
Axial *T*_2_W (A) and STIR (B) images demonstrating stress fractures in the bilateral femur neck as linear hypointense bands with surrounding oedema. STIR, short-tau inversion recovery.

MRI of pelvis with bilateral hip joints demonstrated stress fractures in the bilateral femoral neck as hypointense signals on *T*_1_W images, *T*_2_W images and hyperintense signals on STIR with surrounding sclerosis and oedema (Figures 3 and 4).

**Figure 5.**
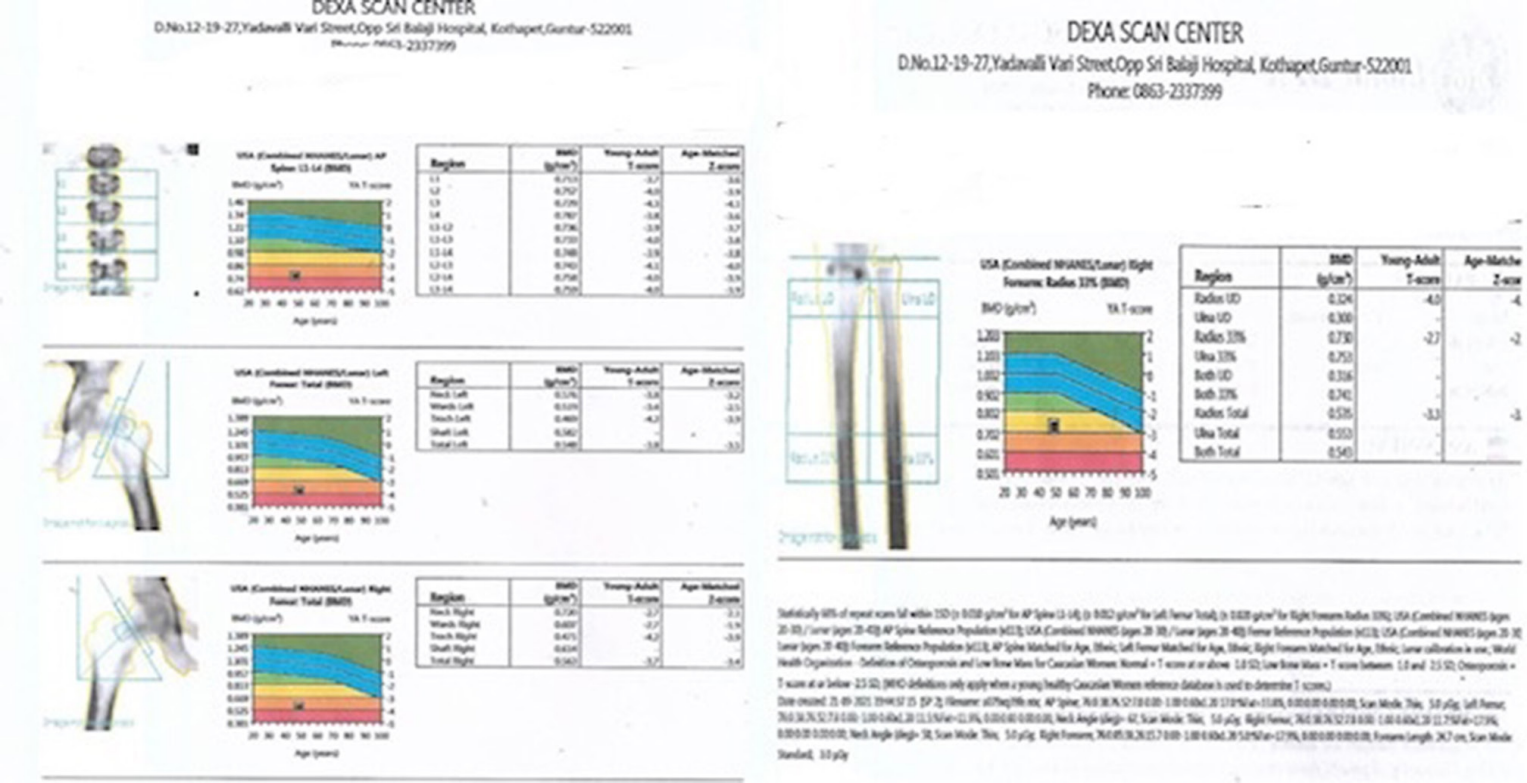
DXA scan done at vertebral, femur and forearm bones demonstrating Tscores and Z scores at different levels suggestive of diffuse osteoporosis of the bones. DXA, dual energy X-ray absorptiometry.

Whole-body bone mineral densitometry (BMD) was performed by Dual energy X-ray absorptiometry (DXA) to assess the mineralisation status of bones (Figure 5). BMD measured at L1–L4 level demonstrated severe osteoporotic changes with the T score value of −3.9 and Z score value of −3.8. At left femur neck level, T score value was −3.8 and z score value −3.5, at right femur neck level, T score was −3.7 and Z score was −3.4. At the level of radius, the T score was −3.3 and Z score was −3.2 (Table 1).

**Table 1.** T score and Z score values of DXA scan report at various levels

LEVEL	T-score	Z-score
L1–L4	−3.9	−3.8
LEFT FEMUR NECK	−3.8	−3.5
RIGHT FEMUR NECK	−3.7	−3.4
RADIUS	−3.3	−3.2

DXA, dual energy X-ray absorptiometry.

### Diagnosis

Based on the history of the patient, clinical presentation and imaging findings, the final diagnosis of bilateral femoral neck stress fractures with severe osteoporosis was considered (Figure 3).

## Treatment and follow-up

Surgical intervention with further medical management and follow-up was advised. As the symptoms were more severe on the left side, surgical intervention by hip arthroplasty was performed, and the femur head was replaced with an Austin-Moore prosthesis. Further conservative management was advised for the right femur fracture. The post-operative period was uneventful. As part of medical management, the patient was advised alendronate sodium tablets (35 mg) once monthly, **vitamin D3 (60,000 IU**) tablets once weekly. He was discharged and was advised for regular follow-up (Figure 4).

## Discussion

High prevalence of decreased BMD has been reported in HIV patients. The exact cause of bone loss in HIV-infected patients was not completely established.

However, multiple factors including generalised physiological decrease in bone turnover, protease inhibitors, nucleoside-related mitochondrial toxicity, lactic acid accumulation, HIV-related lipodystrophy, immune reconstitution, nutritional and hormonal factors, prior AIDS-related wasting, and the damage by the virus itself are implicated as the potential causative factors.^1,2^ Although the use of HAART has dramatically reduced the morbidity and mortality rates from HIV infection, long-term use of HAART results in multiple metabolic disorders including osteopaenia/osteoporosis. Among the drugs used in HAART, protease inhibitors have high potential for causing osteoporosis/osteopaenia.^3^

On review of literature, many studies describing high prevalence of osteopaenia and osteoporosis in HIV patients on HAART regimen were reported (Table 2).

**Table 2.** Review of literature describing the risk and prevalence of osteoporosis/osteopaenia in HIV patients on HAART therapy

AUTHOR	FINDING
**Mondy et al** ^4^	Osteopaenia or osteoporosis was noted in 46% of cases based on the World Health Organisation criteria.
**Knobel et al** ^5^	Osteopaenia was noted in 67.5% of cases and osteoporosis in 21.2% of cases.
**Tebas Pet et al**	50% of cases receiving protease inhibitors had osteopaenia or osteoporosis, compared with 23% of cases not receiving these antiviral drugs.
**Cassetti et al** ^6^	Bone mineral density decreases by 2–6% within the first two years after initiation of various ART regimens.
**Juliet Compston et al** ^7^	Significant bone loss from the spine and hip occurs during the first 1–2 years after the initiation of antiretroviral therapy.

ART, antiretroviral therapy; HAART, highly active antiretroviral therapy.

Although HIV patients are at high risk for stress fractures due to osteoporosis, diagnosis of these fractures are often missed or delayed for several weeks due to lack of symptoms and absence of prior traumatic history. Further incomplete femoral neck stress fractures are often difficult to diagnose on plain radiographs. On review of literature, very few case reports describing bilateral sequential neck fractures of femoral neck have been reported. **Chaganty et al** ^8^ reported a bilateral sequential femoral neck fracture in a 35-year-old HIV patient on ART.

Although radiography is the first line of imaging modality, it seldom demonstrates the fracture. MRI has higher sensitivity in evaluating early stress fractures. **Lassus et al** ^9^ reported radiographs to have a sensitivity of 15–30% for early-stage injuries and 30–70% for late-stage injuries, while MRI is considered to have a near 100% sensitivity at all stages. Although CT is not considered a first/second-line imaging modality, it may have a role where the MRI results are equivocal, owing to high specificity.

The estimation of BMD by DXA scan is used as a screening tool to determine osteoporosis/osteopaenia and increased risk for stress fractures.

Bone scintigraphy identifies insufficiency fractures and pseudofractures early in their course before they are evident on conventional imaging. Bone scintigraphy should be investigation of choice in cases of limb pain, to identify the risk of stress fractures even before their occurrence to prevent further complications.^10^

## Conclusion

HIV infection and ART are risk factors for osteopaenia and osteoporosis and subsequent increased risk of stress fractures. Hence, these patients are advised for regular screening and correction of osteopaenia/osteoporosis ().

## Learning points

- Osteopaenia/osteoporosis are frequently observed in HIV patients on HAART treatment, with increased risk of stress fractures.
- BMD with DXA scan aids in assessment of osteopaenia/osteoporosis.
- MRI is a better diagnostic tool for identification of stress fractures in these patients.
- Regular screening for osteopaenia/osteoporosis helps to prevent the complication of stress fractures in these patients and improve their quality of life.
